# Public Awareness and Perceptions about Diabetes in the State of Qatar

**DOI:** 10.7759/cureus.2671

**Published:** 2018-05-22

**Authors:** Al-Anoud Al-Thani, Aiman H Farghaly, Hammad Akram, Shams Eldin Khalifa, Benjamin Vinodson, Alma M Loares, Abdul-Badi Abou-Samra

**Affiliations:** 1 Ministry of Public Health, State of Qatar, Doha, QAT; 2 Department of Medicine, Hamad Medical Corporation

**Keywords:** diabetes, diabetes awareness, qatar, public perception

## Abstract

Introduction

Diabetes is a well-known global public health challenge affecting millions globally. The aims of this study are to examine the community diabetes knowledge, perceptions, and awareness among the public in Qatar regarding (1) disease symptoms, risk factors, complications, prevention, and associated behaviors, and (2) local diabetes campaigns and available services or resources.

Methods

This study involved a total of 501 respondents selected through purposive sampling from major public malls and public places in Doha, Qatar between February and May 2015. Data were gathered by face-to-face interview utilizing a semi-structured questionnaire. Results were analyzed using count, percentage, median, chi-square test, z-test on proportion and logistic regression. The analysis was carried out at 5% level of significance using SPSS version 22 (IBM Corporation, Chicago, IL, USA).

Results

About 92% of participants knew at least one type of diabetes. Over 43.9% were physically active for 1–3 days per week. Highest proportion of the population perceived blindness (86%) as the top complication; frequent urination (58%) and excessive thirst (41%) as primary symptoms; hereditary factors (74%) and obesity (74%) as the top risk factors; and exercise (77%) and diet (72%) as among the preventive measures. Demographic overview of diabetes and lifestyle factors showed that the odds of obtaining screening tests were higher among females (OR=1.7, P=0.003), age 35–45 (OR=2.2, P=0.003), age ≥55 (OR=4.1, P<0.001) and married (OR=3.0, CI=2.0–4.6, P<0.001) compared with their respective counterpart groups.

Conclusions

The general population in Qatar require more awareness and education about diabetes prevention and associated risk factors. This could be achieved by implementation of more public health campaigns to encourage healthier lifestyles.

## Introduction

Diabetes is a well-known global public health challenge affecting over 400 million people worldwide. Despite the awareness of the problem and technological advancements in healthcare systems, the adult diabetes prevalence has almost doubled since 1980 worldwide. Diabetes is a huge financial burden because of the associated multi-organ complications among sufferers and a major cause of mortality around the world [1]. The Arab world is not spared by the diabetes epidemic as well, for example in the Eastern Mediterranean region countries the prevalence of this disease ranges from 3.5% to as high as 30% [2].

The State of Qatar is a country located in the Gulf Cooperation Council region adjacent to Arabian Gulf with over 2.5 million population. The country has encountered a rapid socio-economic development in recent past few decades that led to a change in lifestyles of the population influenced by things such as easily available fast food, increased use of personal vehicles for transportation, increased screen time and fewer chances of being physically active etc. Diabetes and associated risk factors such as obesity and lack of physical activity are now considered major public health challenges in Qatar [3-4].

Qatar’s National Health Strategy (NHS) envisions to encourage a healthy lifestyle, promote public health and ensure that quality healthcare services are accessible to the community [5]. With the implementation of one of its strategies, the National Diabetes Strategy, which aims to increase public awareness about diabetes, promote prevention and support healthy lifestyles, NHS aspires to decrease the incidence and complications of diabetes for better health and quality of life in Qatar [6]. This is important since according to a national survey in Qatar that was conducted in 2012, the diabetes prevalence among Qatari nationals was 16.7% which is higher than the prevalence identified in a 2006 survey. In the same survey, 11.6% Qatari nationals, 6.6% non-Qatari residents composed of Arab, Asian and Western nationals, and 8.3% of the total sample had self-reported diabetes or were treated with medicines for it [7-‎8]. The survey results have also indicated that certain behavioral and social factors have an impact on obesity as well as on diabetes prevalence among population [7, 9].

In 2015, the Ministry of Public Health (MoPH), Qatar with the assistance of YouGov (a market research organization) performed a public survey named “Diabetes Awareness and Perception in Qatar” to assess the knowledge of community pertaining to diabetes and associated factors. The survey was an important initiative to further strengthen the existing national strategy that addresses various non-communicable diseases (NCDs) and promote prevention, monitoring and community education related to diabetes as well.

The present study provides an overview of the results of the above-mentioned survey and examines the community perception and awareness about diabetes and related factors such as diabetes symptoms, risk factors, complications, prevention, and lifestyle behaviors. The study further explores the relationship of demographic factors with diabetes screening and selected behavioral factors.

## Materials and methods

Sampling

The study was carried out at public places with the general population, such as malls and shopping centers. A total of 501 individuals were interviewed face-to-face for about 20–30 minutes between February and May 2015. This sample provided a margin of error of ±4.4% at 95% confidence interval. The target population was 16 years or older and included both genders from different nationalities categorized as Qatari, Arab expats, Asian expats, and Western expats. The sample selection was achieved by using purposive sampling method, with inclusion criteria based on quotas per gender, age groups, and nationality (Table 1). Interviews were conducted as per accordance with the sampling plan in which an equal number of Qatari and non-Qatari citizens and an equal number of male and female participants were selected. Age groups were categorized into 16–24 years, 25–34 years, 35–54 years and 55 years or older (>54). The sampling proportion of each age group was selected to correspond approximately to the estimated 2015 Qatar census proportion within the same age groups. A sample of 501 was achieved based on the availability of budget, resources and associated logistics.

**Table 1:** Proposed Sample Quota for Survey, By Gender, Nationality and Age Groups

	Qataris		Expats		Total
Age	Males	Females	Males	Females	
16 – 24	25	25	20	15	85
25 – 34	40	40	60	35	175
35 – 54	40	40	60	35	175
>54	20	20	15	10	65
TOTAL	250		250		500

In addition to the demographic and socio-economic information of participants, the survey questions were designed to obtain their perception, awareness and knowledge related to diabetes. The participants were able to share their awareness about local campaigns and the mode of related messages transmitted to them, knowledge about the types, risk factors, symptoms, complications of diabetes and understanding of preventive factors. Furthermore, the survey also covered participant’s personal behaviors in terms of physical activity and diet.

Analysis

Statistical analyses were performed using the SPSS software version 22.0 (IBM Corporation, Chicago, IL, USA). The results were reported as sample size and/or percentage with 95% confidence interval while awareness about complications of diabetes was measured using a 5-point Likert scale responses [1–strongly disagree, 2– disagree, 3–neither disagree nor agree (neutral), 4–agree, 5–strongly agree] and generally assessed using median value. Chi-square test was performed to determine the association between gender, and variables assessing diabetes awareness and healthy lifestyle. Again, Z test on proportion was used to evaluate the differences in proportions by gender. The effect of various demographic factors on diabetes screening, frequencies of exercises, and healthy eating habits was assessed by logistic regression, which computed odds ratio and 95% confidence interval. Level of significance was given at P<0.05.

Ethical procedures

Ethical procedures were followed during the survey implementation and data handling procedures. The consent was obtained from the participants before the survey was carried out. The collected information was only used for the scientific purposes without breaching any personal or confidential information. Parental consent was sought for the respondents aged below 18 years.

## Results

Characteristics of Participants

A total of 501 participants of ages 16 years or older were interviewed. Based on the selection criteria, about equal numbers of Qatari and non-Qatari nationals were included (48% and 52%, respectively); and about equal numbers of male and female subjects (51.7% and 48.3%, respectively). Highest number of participants were in the age group 24–34 years (170, 33.9%) followed by 35–54 years (161, 32.1%), 16–24 years (110, 22%) and 55+ (60, 12%) respectively. About one-half of all participants (50.9%) reported being married with children. The characteristics of participants are shown in Table 2.

**Table 2:** Characteristics of Study Population

General characteristics	
N	501
	n (%)
Age	
16-24 years	110 (22.0)
25-34 years	170 (33.9)
35-54 years	161 (32.1)
55+ years	60 (12.0)
Gender	
Male	259 (51.7)
Female	242 (48.3)
Nationality group	
Qatari	239 (47.7)
Arab	108 (21.6)
Asian	97 (19.4)
Westerner	57 (11.4)
Married status	
Single	179 (35.7)
Married with children	255 (50.9)
Married without children	51 (10.2)
Widowed/ divorced	16 (3.2)
Monthly Income	
1,500-9,999 QAR	96 (19.2)
10,000- 19,999 QAR	128 (25.5)
20,000+ QAR	214 (42.7)
Don’t know/ can’t say	63 (12.6)

Diabetes Awareness and Screening

The respondents were asked if they were aware of different types of diabetes. The first type of diabetes reported by each respondent was recorded under one variable. Then the second response was recorded as a separate variable using the question “What other types of diabetes are you aware of” (data not shown in this article). As a first response, 92% were able to provide a type of diabetes while 38 (7.6%) stated that they did not know any type(s). A total of 61 (12.2%) respondents stated that they were diagnosed with diabetes (10% men, 14.5% women, 17% Qatari, 8% non-Qatari), 6.2% also had someone in their family or friends with diabetes, and about 45% stated that someone close to them had been diagnosed with diabetes (Table 3).

**Table 3:** Participant’s Awareness Pertaining to Diabetes

Diabetes Awareness	Total n (%)	Men n (%)	Women n (%)
N	501	259	242
Diagnosed with diabetes			
Diagnosed with diabetes (myself)	61 (12.2)	26 (10.0)	35 (14.5)
Diagnosed with diabetes myself and members of family/ friends	31 (6.2)	18 (6.9)	13 (5.4)
Someone close to me who has been diagnosed with diabetes	225 (44.9)	107 (41.3)	118 (48.8)
Neither I nor family / friends have been diagnosed with diabetes	184 (36.7)	108 (41.7)	76 (31.4)
Awareness based on type of diabetes			
Pre-diabetes	131 (26.1)	78 (30.1)	53 (21.9)‡
Type 1 Diabetes	204 (40.7)	117 (45.2)	87 (36.0)
Type 2 Diabetes	69 (13.8)	43 (16.6)	26 (10.7)
Gestational diabetes	59 (11.8)	5 (1.9)	54 (22.3)
None of the above	38 (7.6)	16 (6.2)	22 (9.1)
Ever Screened for Diabetes			
Yes	184 (36.7)	79 (30.5)	105 (43.4)‡
No	317 (63.3)	180 (69.5)	137 (56.6)
Ways to prevent Diabetes ^a^			
Frequent exercise	386 (77.0)	208 (80.3)	178 (73.6)
Healthy Diet	363 (72.5)	199 (76.8)	164 (67.8)*
Maintain a healthy weight	301 (60.1)	150 (57.9)	151 (62.4)
Get checked	302 (60.3)	152 (58.7)	150 (62.0)
Quit smoking	100 (20.0)	60 (23.2)	40 (16.5)
Top 5 perceived signs of diabetes ^a^			
Frequent urination, particularly at night	291 (58.1)	158 (61.0)	133 (55.0)
Excessive thirst	205 (40.9)	112 (43.2)	93 (38.4)
Tiredness	174 (34.7)	87 (33.6)	87 (36.0)
Distorted vision	162 (32.3)	84 (32.4)	78 (32.2)
Tingling/ Numbness of hands and feet	152 (30.3)	77 (29.7)	75 (31.0)
Preferred location for diabetes check in case of signs of diabetes^a^			
Public Hospital	281 (56.1)	145 (56.0)	136 (56.2)
Private hospital	178 (35.5)	103 (39.8)	75 (31.0)*
Private clinic	147 (29.3)	92 (35.5)	55 (22.7)*
Diabetes care center	203 (40.5)	110 (42.5)	93 (38.4)
As home with a home test kit	141 (28.1)	62 (23.9)	79 (32.6)*
Perceived top risk factors of diabetes ^a^			
Hereditary	370 (73.9)	200 (77.2)	170 (70.2)
Injury or disease of the pancreas & insulin	314 (62.7)	162 (62.5)	152 (62.8)
Obesity	370 (73.9)	198 (76.4)	172 (71.1)
Lack of exercise	309 (61.7)	164 (63.3)	145 (59.9)
Poor Diet	307 (61.3)	163 (62.9)	144 (59.5)
High blood glucose	305 (60.9)	159 (61.4)	146 (60.3)
Top 5 sources of ad or campaign about diabetes ^a^			
TV ad	54 (35.3)	28 (35.9)	26 (34.7)
Social media	32 (20.9)	18 (23.1)	14 (18.7)
Internet	30 (19.6)	16 (20.5)	14 (18.7)
Billboard	60 (39.2)	33 (42.3)	27 (36.0)
At public hospital/ clinic	38 (24.8)	20 (25.6)	18 (24.0)
Organizations communicated diabetes awareness			
Action of diabetes	18 (11.8)	10 (12.8)	8 (10.7)
Qatar Diabetes association	34 (22.2)	16 (20.5)	18 (24.0)
Ministry of Public Health	44 (28.8)	23 (29.5)	21 (28.0)
Major Community Hospital (Hamad Medical Corporation)	51 (33.3)	29 (37.2)	22 (29.3)
*- indicates proportion is significantly different from men. ‡- indicates distribution of proportions is significantly different betweengender at 5% level. ^a - ^multiple response			

**Figure 1:**
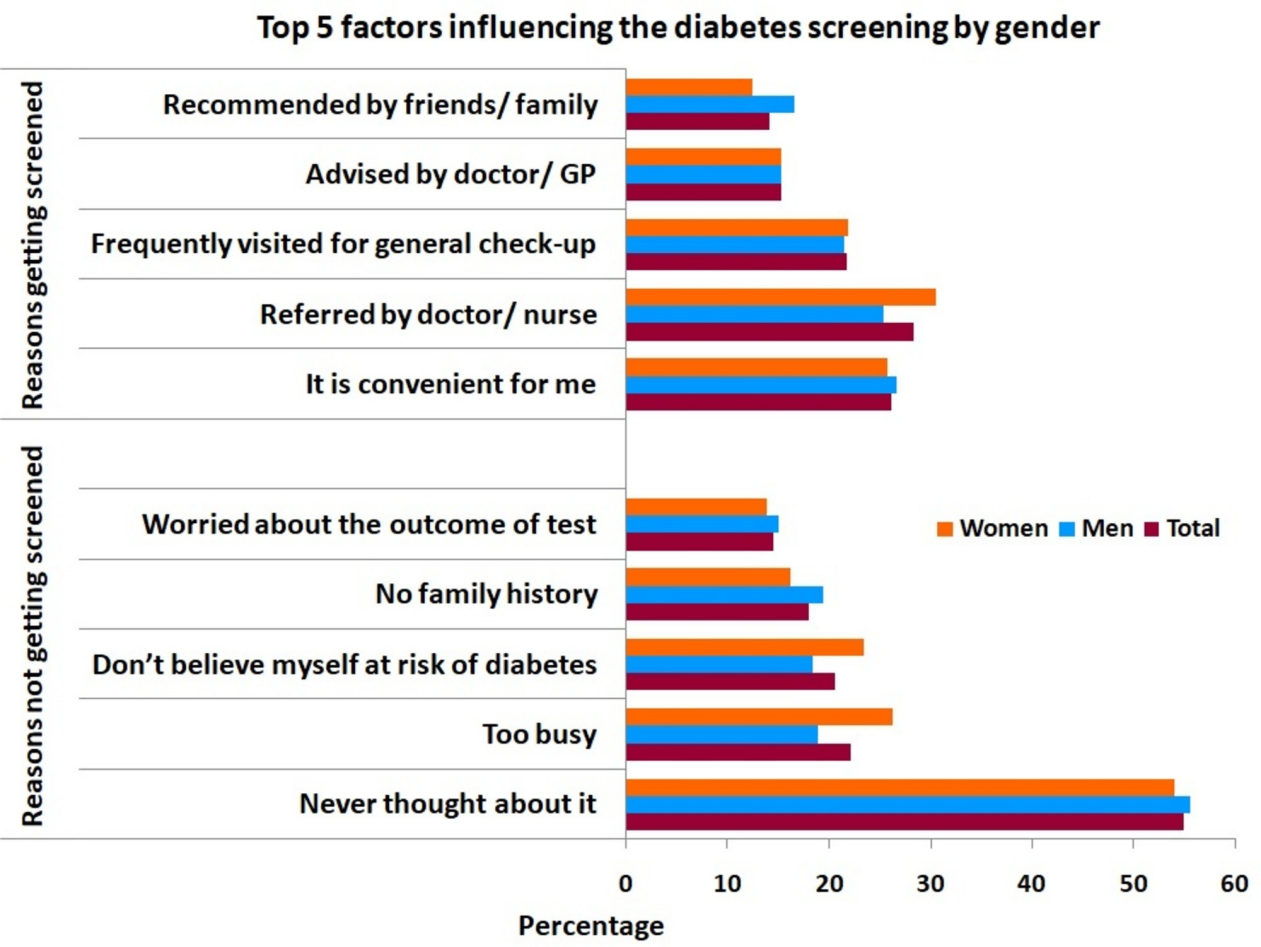
Top 5 Influencing Factors for Diabetes Screening Among Participants by Gender

**Figure 2:**
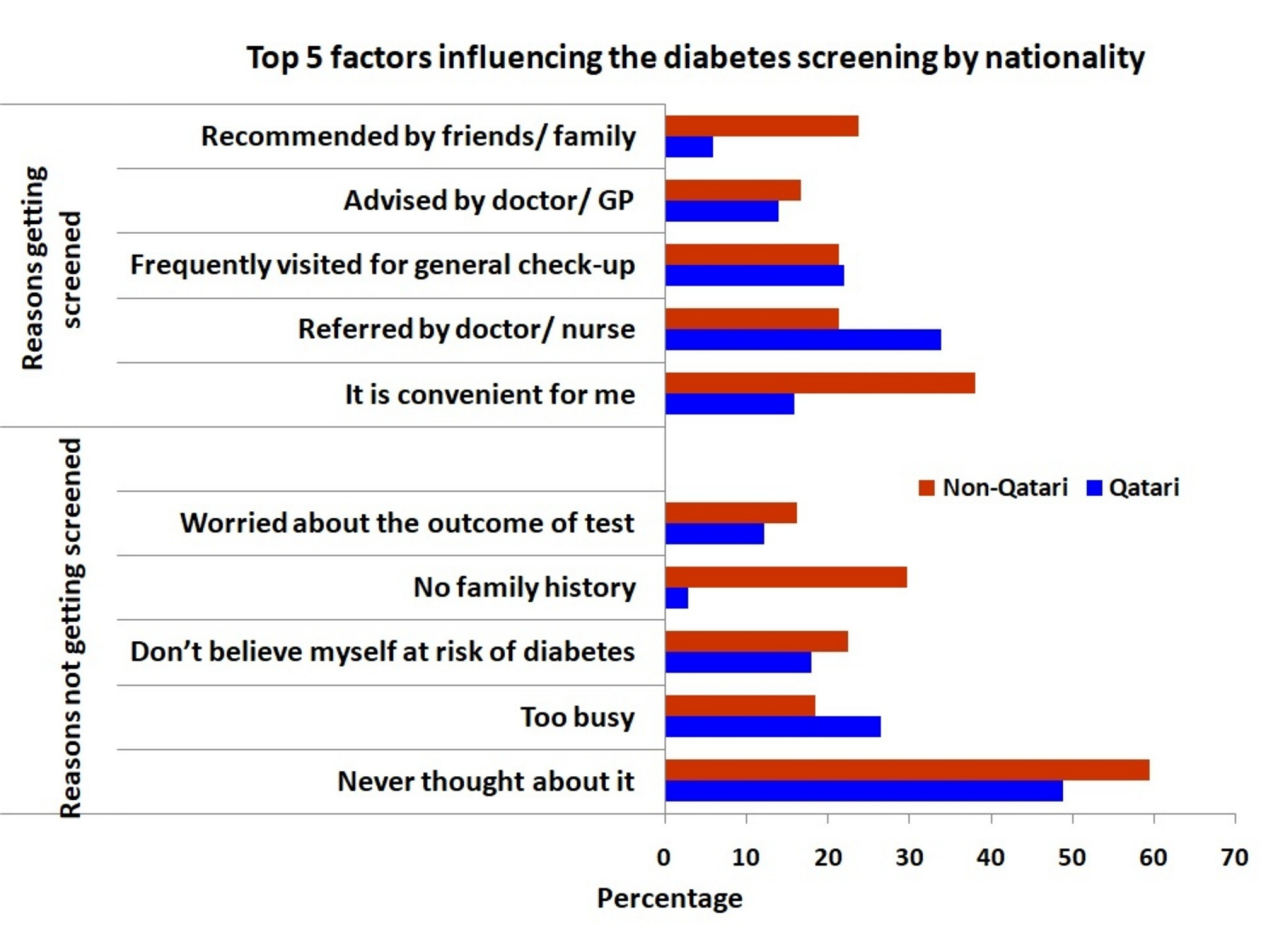
Top 5 Influencing Factors for Diabetes Screening Among Participants by Nationality

Overall, 36.7% stated that they were screened for diabetes with a higher percentage among women (43.4%) compared with men (30.5%). Most common location to obtain diabetes screening was diabetes clinic (34.2%) followed by the primary health care centers (29.3%). The top reason to obtain screening was physician or nurse referral (28%) followed by the convenience of getting screening (26%) (Figure 1). Among Qatari nationals, the top reason for obtaining screening (34%) was the referral from a doctor or a nurse. For non-Qataris, the top reason for getting screening was family or friends recommendations (23.8%). Those who were not screened, 59.6 Qatari and 48.9% non-Qatari, stated that the main reason for not obtaining screening was that they never thought about it (Figure 2). This was also the main reason for the overall sample (Figure 1). When participants were asked if they know the ways to prevent diabetes, the majority considered the frequency of exercise (77%) followed by healthy diet (72.5%) and maintaining a healthy weight (60%) as preventive measures (Table 3). Regarding the signs and symptoms of diabetes, over 58% of participants mentioned the frequency of urination especially at night is the main sign followed by excessive thirst (41%) and tiredness (34.7%). The respondents considered obesity (73.9%) and hereditary factors (73.9%) as the top risk factors in the development of diabetes followed by almost equal responses (60%) for pancreatic disorders, poor diet, lack of exercise, getting older, and high blood glucose. Billboards (39.2%) were considered by respondents as the main source of diabetes campaigns advertisement followed by television (35.3%) (Table 3).

Perceptions about diabetes and respondents’ awareness on diabetes complications were likewise determined as shown in Table 4. Except for one, all of the questions evaluating the diabetes awareness derived a median value of 4, which indicates that half of the respondents agreed to the statements mentioned. Salient to the findings was that majority of the participants had highest awareness on blindness as a complication of diabetes, as more than half strongly agreed that diabetes causes blindness, having a median value of 5.

**Table 4:** The Respondent's Perceptions of Diabetes and Related Complications

	Strongly disagree % (CI%)	Disagree % (CI%)	Neutral % (CI%)	Agree % (CI%)	Strongly agree % (CI%)	Median
Advanced screening tools available to detect diabetes	0.4 (0.1, 1.4)	1.0 (0.4, 2.3)	23.2 (19.7, 27.0)	31.5 (27.6, 35.7)	43.9 (39.6, 48.3)	4
People with diabetes have a higher risk of developing infections	2.0 (1.0, 3.6)	7.4 (5.4, 10.0)	26.1 (22.5, 30.1)	48.3 (43.9, 52.7)	16.2 (13.2, 19.6)	4
Diabetes is a leading cause of CVD	3.9 (2.6, 6.1)	8.6 (6.4, 11.4)	20.8 (17.4, 24.5)	31.7 (27.8, 35.9)	34.9 (30.8, 39.2)	4
Diabetes can cause blindness	0.2 (0.04, 1.1)	1.0 (0.4, 2.3)	12.4 (9.8, 15.6)	28.3 (24.6, 32.4)	58.1 (53.7, 62.3)	5
Kidney failure is a serious complication of diabetes	0.4 (0.1, 1.4)	4.6 (3.1, 6.8)	25.9 (22.3, 29.9)	28.9 (25.1, 33.1)	40.1 (35.9, 44.8)	4
People with diabetes carry a high risk of amputation	0.4 (0.1, 1.4)	8.8 (6.6, 11.6)	19.8 (16.5, 23.5)	38.5 (34.4, 42.8)	32.5 (28.6, 36.7)	4
Diabetes can cause pregnancy complications in women with any type of diabetes	0.2 (0.04, 1.1)	3.4 (2.1, 5.4)	23.8 (20.2, 27.7)	37.7 (33.6, 42.0)	34.9 (30.9, 39.2)	4
Type 1 diabetes cannot be prevented	0.4 (0.1, 1.4)	7.2 (5.2, 9.8)	28.1 (24.4, 32.2)	30.5 (26.7, 34.7)	33.7 (29.7, 37.9)	4
Healthy life-style choices can help prevent type 2 diabetes	0.6 (0.2, 1.8)	4.2 (2.7, 6.3)	20.8 (17.4, 24.5)	36.1 (32.0, 40.4)	38.3 (34.2, 42.6)	4
Blood sugar should be checked at least once a year	8.4 (6.2, 11.1)	7.0 (5.1, 9.6)	19.0 (15.8, 22.6)	39.9 (35.7, 44.3)	25.7 (22.1, 29.8)	4
* Percentage and 95% Confidence Intervals are shown in the table. ​​​​​​​						

Lifestyle

Over 43.9% of the respondents were physically active for 1–3 days per week, 31.5% were engaged in it 2–3 times per week and only 11% have a daily physical activity. Furthermore, 25% stated that they do not perform any exercise throughout the week. Most common place of the exercise was fitness centers (31%) followed by public areas (21%), homes (17%) and sports clubs (15%), respectively. About 57% of respondents considered their eating habits as healthy and 59.7% were satisfied or very satisfied with their eating habits. The family’s persuasion had the highest impact on the change in eating habits of the respondents (59.7%) followed by doctor’s advice (48.9%). The respondents stated that in order to keep a track of their health they measure their body weight (61.5%), monitor calorie intake (43.5%), assess fitness level (37.1%) and/or use a health/fitness mobile application (29.1%). On the contrary, about 7% do not track their health (Table 5).

**Table 5:** Lifestyle Attributes of Survey Participants

Healthy lifestyle attributes	Total n (%)	Men n (%)	Women n (%)
	501	259	242
Frequency of exercise (C1)			
No exercise	125 (25.0)	56 (21.6)	69 (28.5)
1-3 times a week	220 (43.9)	123 (47.5)	97 (40.1)
4-7 times a week	156 (31.1)	80 (30.9)	76 (31.4)
Place of exercise (C2)^a^			
Fitness center/ Gym	118 (31.0)	75 (36.9)	43 (24.8)
Public areas	79 (21.0)	41 (20.2)	38 (22.0)
Sports club	55 (15.0)	28 (13.8)	27 (15.6)
At home	64 (17.0)	27 (13.3)	37 (21.4)
Doha Corniche (city beachline)	34 (9.0)	18 (8.9)	16 (9.2)
Public parks	24 (6.4)	13 (6.4)	11 (6.4)
Consider Eating habits healthy? (C3)			
Yes	285 (56.9)	152 (58.7)	133 (55.0)
No	181 (36.1)	89 (34.4)	92 (38.0)
Don’t know	35 (7.0)	18 (6.9)	17 (7.0)
Satisfied with current eating habits (C4)			
Very satisfied	101 (20.2)	53 (20.5)	48 (19.8)
Satisfied	198 (39.5)	112 (43.2)	86 (35.5)
Neither satisfied or dissatisfied	162 (32.3)	77 (29.7)	85 (35.1)
Dissatisfied	29 (5.8)	13 (5.0)	16 (6.6)
Very dissatisfied	11 (2.2)	4 (1.5)	7 (2.9)
Top 5 factors/ persons who can persuade you to change eating habits (C5)^a^			
Family	299 (59.7)	151 (58.3)	148 (61.2)
Doctor	245 (48.9)	115 (44.4)	130 (53.7)*
Self	187 (37.3)	107 (41.3)	80 (33.1)
Friends	165 (32.9)	85 (32.8)	80 (33.1)
Dietician	109 (21.8)	49 (18.9)	60 (24.8)
Ways to keep track of health (C6)^a^			
Weight	308 (61.5)	157 (60.6)	151 (62.4)
Counting steps	117 (23.4)	68 (26.3)	49 (20.2)
Monitoring calorie intake	218 (43.5)	108 (41.7)	110 (45.5)
Fitness level	186 (37.1)	111 (42.9)	75 (31.0)*
Health/ Fitness app	146 (29.1)	85 (32.8)	61 (25.2)
Don’t track health	34 (6.8)	14 (5.4)	20 (8.3)
*- indicates proportion is significantly different from men. ‡- indicates distribution of proportions is significantly different between gender. ^a - ^multiple response			

The relationship of demographic factors and lifestyle was evaluated by calculating odds ratios. The results showed that the participants with older age groups, 35 to 45 years, and 55 years and higher in comparison to the individuals in age groups 16 to 24 years, were more likely to have had a diabetes screening test with the odds ratios of 2.2 (CI=1.3–3.7, P=0.003) and 4.1 (CI=2.1–8.2, P<0.001); respectively. Females were more likely to receive screening compared with males (OR=1.7, CI=1.2–2.5, P=0.003) (Table 6). Furthermore, married (OR=3.0, CI=2.0–4.6, P<0.001) and widowed/divorced participants (OR=3.7, CI=1.3–10.5, P =0.01) were more likely to obtain screening compared to singles. It was also found that the females were less likely to be physically active compared with men who were active 1–3 times a week (OR=0.64, CI=0.41-0.99, P=0.04). Qatari citizens were less physically active in both categories i.e. 1–3 times/wk (P <0.001) and 4–7 times/wk (P<0.001) compared with non-Qatari participants. Furthermore, Qatari nationals were also less likely to have healthy eating habits (OR=0.29, CI=0.19-0.43, P<0.001) compared with non-Qataris (Table 6). The participants who were physically active 4–7 times per week (OR=0.20, CI=0.08–0.49, P<0.001) and eating healthy (OR=0.45, CI=0.24–0.82, P=0.009) were less likely to report that they were diagnosed with diabetes.

**Table 6:** Relationship of Demographic Factors with Lifestyle and Diabetes Diagnosis

Profile	Screened for diabetes		Frequency of exercise				Healthy eating	
			1-3 times a week		4-7 times a week			
	OR (95 % CI)	P value	OR (95 % CI)	Pvalue	OR (95 % CI)	P value	OR (95 % CI)	P value
Age groups								
16-24	Ref.		Ref.		Ref.		Ref.	
25-34	1.1 (0.64, 1.9)	0.73	0.56 (0.29, 1.1)	0.07	0.79 (0.40, 1.5)	0.48	0.97 (0.58, 1.6)	0.92
35-54	2.2 (1.3, 3.7)	0.003*	0.64 (0.34, 1.2)	0.17	0.50 (0.25, 1.0)	0.05*	0.93 (0.56, 1.5)	0.79
55+	4.1 (2.1, 8.2)	<0.001*	0.85 (0.37, 1.9)	0.7	0.85 (0.35, 2.1)	0.72	1.2 (0.61, 2.3)	0.6
Gender								
Male	Ref.		Ref.		Ref.		Ref.	
Female	1.7 (1.2, 2.5)	0.003*	0.64 (0.41, 0.99)	0.04*	0.77 (0.48, 1.2)	0.28	0.84 (0.58, 1.2)	0.38
Nationality								
Qatari	1.5 (1.1, 2.2)	0.02*	0.31 (0.19, 0.49)	<0.001*	0.34 (0.21, 0.56)	<0.001*	0.29 (0.19, 0.43)	<0.001*
Non-Qatari	Ref.		Ref.		Ref.		Ref.	
Marital status								
Married	3.0 (2.0, 4.6)	<0.001*	0.73 (0.45, 1.1)	0.19	0.58 (0.35, 0.97)	0.04*	1.1 (0.77, 1.7)	0.5
Single	Ref.		Ref.		Ref.		Ref.	
Widowed/ divorced	3.7 (1.3, 10.5)	0.01*	0.64 (0.18, 2.1)	0.46	0.45 (0.11, 1.8)	0.25	0.68 (0.24, 1.9)	0.46
Income group								
1,500-9,999 QAR	0.41 (0.23, .70)	0.001	7.4 (3.2, 17.3)	<0.001*	4.9 (2.0, 11.9)	<0.001*	4.7 (2.5, 8.9)	<0.001*
10,000- 19,999 QAR	0.57 (0.35, .91)	0.01	1.9 (1.1, 3.4)	0.01*	1.1 (0.64, 2.2)	0.58	1.1 (0.74, 1.8)	0.47
20,000+ QAR	Ref.		Ref.		Ref.		Ref.	
Diagnosed with Diabetes(B1)								
Yes			0.79 (0.40, 1.5)	0.49	0.20 (0.08, 0.49)	<0.001*	0.45 (0.24, 0.82)	0.009*
No			Ref.		Ref.		Ref.	
* significant at 5% level Reference for Frequency of Exercise: Without exercise								

## Discussion

The State of Qatar’s National Health Strategy considers diabetes as one of the high-priority diseases for preventive healthcare [5]. Our study aimed at assessing the awareness and perception among the Qatar population including Qatari nationals and expatriates. Increased awareness among the population is a powerful step towards prevention of diabetes as it helps to assess causes, risks and complications and prompt people at risks to seek treatment and care.

The study revealed that about 92% of respondents knew about at least one type of diabetes. Our results may not be comparable to other studies due to the unique methodology and location; however, in somewhat similar Indian study about 75% of the respondents had general awareness about diabetes [10]. In another study from an Ethiopian community showed that about 52.5% of the study participants were knowledgeable about diabetes [11]. Moreover, two other studies describe lack of knowledge pertaining to diabetes for example; a study conducted in Malaysia showed that around 58.1% of respondents had poor diabetes knowledge score among rural adult community [12]. Another Indian study, involving outpatients and inpatients showed that about 46% of diabetic patients had poor knowledge whereas interestingly 64% of non-diabetics had good knowledge regarding diabetes [13].

While examining the perceptions about risk factors leading to diabetes, majority of respondents considered hereditary factors (73.9%), obesity (73.9%) followed by pancreatic disease/injury (62.7%), lack of exercise (61.7%) and poor diet (61.3%) as causative reasons in the development of the disease. These findings are somewhat consistent with the results of an Indian study where respondents considered family history of diabetes, consumption of more sweets, lack of exercise and obesity as main reasons of diabetes [10]. This is also congruent to the survey results done in a semi-urban Omani population, which identified obesity, physical inactivity and family history of diabetes as the top risk factors of diabetes perceived by the respondents [14]. In Qatar’s case of T2DM for instance, a recent forecast of diabetes in Qatar highlighted obesity as the main driver of the rising diabetes prevalence in the country. About 60% of type 2 diabetes mellitus (T2DM) burden was ascribed to obesity while 10% was attributed to smoking and physical inactivity combined [15].

While examining respondents’ awareness about complications of diabetes, blindness was highly perceived as the most common outcome. Moreover, the majority of respondents agreed upon the fact that diabetes could cause amputation, kidney problems, and cardiovascular disease with a median response of 4 for these conditions. This finding is congruent to a study conducted in Oman whereby respondents identified the visual problem as the top complication, with heart disease, kidney disease, and wound infections as other complications [14]. In other studies, the respondents also identified foot problems, kidney disease, eye disease and cardiovascular diseases as complications of diabetes which is somewhat consistent with our findings [14, 16].

In our study, 12% of responded stated that they had diabetes with a higher proportion among females and Qatari nationals. This finding is comparable with the other existing data and studies from Qatar [4, 7, 9]. Moreover, the results from 2012 STEPwise survey in Qatar showed that about 12.7% of respondents were diagnosed with diabetes by a doctor or other health worker in past 12 months which is very close to our finding [17].

One of the challenges identified in our study was a relatively low screening proportion among participants and the lack of awareness about it. The results indicated that the lack of screening was mainly attributed to the fact that participants never thought about it. The main reasons for obtaining screening were a referral by health care provider and convenience of the process. Participants of older age groups, females, and people who were ever married were found more likely to be screened compared to their counterpart groups. This could be attributed to the fact that age is a risk factor for diabetes and females are mostly screened for gestational diabetes at the time of their first prenatal visit especially if they have existing risk factors. Gestational diabetes, which affects an estimated 18 percent of women during pregnancy, is caused by a change in the way a woman's body responds to insulin during pregnancy could be a major risk factor for the development of T2DM [18]. According to Kim et al, the only factors consistently associated with better screening rates were marital status and visit with an endocrinologist after delivery which increases the chances of screening among mothers especially who are perceived to be at higher risk [‎19]. Failure to screen for T2DM could result in missed opportunities for disease prevention [19]. Moreover, McCaig highlighted that marriage can provide a positive encouragement or support for a partner to be health conscious and to practice a healthy lifestyle [20]. 

In this study, the public hospital system was identified as the most preferred location for diabetes care in situations where respondents had any associated symptoms. This shows the important role and ease of accessibility of public sector health services in Qatar. The State of Qatar provides health services to all citizens and residents through government medical facilities through a health card system [21]. The system allows residents and citizens to obtain health services at various public healthcare facilities at no cost or at subsidized rates depending upon the level of care. Furthermore, to support the rising encounters in the health sector an increase in the number of healthcare facilities and workforce has been prioritized [22]. This effort ensures sufficient capacity in the system and health infrastructure in place in the future.

An important finding of this study was that the physical activity was relatively low with 25% of respondents reporting that they did not perform any exercise. The proportion of low physical activity was higher among female participants and consistent with the findings from previously completed studies in Qatar and regionally in Kuwait [4, 7, 23]. It was also evident that the Qatari nationals and females (Qatari and non-Qatari) were less likely to be physically active compared to non-Qataris and males (Qatari and non-Qatari), respectively. About 57% of respondents considered their eating habits healthy, 60% were satisfied or very satisfied with their eating habits. The family persuasion was the major factor in changing eating habits as evident by another study, which supports the fact the family and social environments could play an important role in the determination of dietary patterns and quality among children [24]. In our study, the people who were physically active and eating healthy were less likely to have had diabetes. This provides evidence that lifestyle modifications such as physical activity can delay the onset or prevent diabetes. To improve public health and further understand the underlying mechanisms behind findings of this study, the surveillance and mixed method approaches (quantitative and qualitative) can be used to study chronic diseases including diabetes and related risk factors especially the ones already exist in Qatar [4, 8-9, 25-28]. 

## Conclusions

One implication of this study is that the general population in Qatar require more awareness and education regarding diabetes prevention and risk factors. Behaviors towards diabetes diagnosis and prevention vary by demographic attributes. It is more likely for older age group, females, Qatari nationals, and married/widowed/divorced to get screened for diabetes; more likely for males, non-Qataris, younger and single people to indulge in exercise; and more likely for non-Qataris to practice healthy eating habits than their counterparts. This demonstrates a need to implement more campaigns to encourage healthier lifestyles. The findings of this study will specifically useful for the Qatar's National Diabetes and Health strategies in further strengthening ongoing and future programs through evidence based approaches.
